# Effects of Pilates on Body Posture: A Systematic Review

**DOI:** 10.1016/j.arrct.2024.100345

**Published:** 2024-05-21

**Authors:** Fangyi Li, Roxana Dev Omar Dev, Kim Geok Soh, Chen Wang, Yubin Yuan

**Affiliations:** Department of Sports Studies, Faculty of Educational Studies, Universiti Putra Malaysia, Serdang, Selangor, Malaysia

**Keywords:** Body posture, Pilates, Pilates method, Poor posture, Posture, Rehabilitation

## Abstract

**Objective:**

To perform a systematic review of the effects of Pilates on common body postures.

**Data sources:**

Web of Science, PubMed, Scopus, Science Direct, Springer Link, and CNKI. The search year is set from January 1, 2019, to November 15, 2023.

**Study Selection:**

Quasi-experimental studies, randomized controlled trials, randomized clinical trials, and nonrandomized controlled trials investigating the effects of Pilates on body posture.

**Data Extraction:**

The Physiotherapy Evidence Database scale was used to evaluate the quality of studies that met the inclusion requirements. Studies were independently assessed by 2 reviewers who read through the full text and labeled as “low quality,” “moderate quality,” “good quality,” and “excellence quality.” Disagreements were resolved by the third reviewer. The Cochrane Risk of Bias (RoB 2.0) tool was used to assess the risk of bias for each study.

**Data Synthesis:**

Of the 492 studies screened, 13 met the inclusion criteria involving a total of 783 trial participants. Six studies (46%) were of high quality or above, with main limitation related to the internal validity of the study design. The research outcomes focused primarily on effects of Pilates on body posture; cervical, thoracic, and lumbar spine; and followed by quality of life and pain.

**Conclusions:**

The findings of this systematic review provided valuable evidence for the role of Pilates in improving body posture problems. Pilates is a boon to patients suffering from postural disorders, and it is suggested that Pilates can be widely used as a complementary therapy. Nonetheless, more detailed studies are necessary in the future.

Postural problems have become one of the most common, yet underestimated, health problems globally.^1^ Most postural problems refer to incorrect body posture in which the body is unable to maintain the normal functioning of tissues and organs in an upright position.^2^ Human body posture undergoes considerable variability, which depends on age, sex, body growth, environmental factors, and psychophysical status of an individual.^3^^,^^4^ Evidence suggests that 30%-50% of children and adolescents have varying degrees of postural problems,^5^ with the most prevalent postural problems being shoulder asymmetry, head forward, thoracic kyphosis, and scapular tilt.^6^ Postural problems carry the risk of a range of unhealthy manifestations, such as pain^7^ and scoliosis.^8^ Literature suggests that heavy school bags can lead to rounded shoulders and hunched backs in children and adolescents^9^ and that prolonged sedentary behavior increases the prevalence of poor body posture as well as back pain in women.^10^ According to our survey, the prevalence of postural problems shows variability in different countries and regions, with varied influencing factors.^5^, ^6^, ^7^^,^^11^, ^12^, ^13^

Physical activity plays a vital role in human health. Regular physical activity is good for the well-being of the body at any age.^14^ Lack of exercise raises one's risk of disease and undesirable conditions, and body posture deformities are one of them.^15^ There are also many means of correcting posture; in addition to traditional medical treatments, precise physiotherapy and the development of a detailed exercise program show their benefits in improving postural problems.^13^^,^^16^, ^17^, ^18^ Pilates exercise was founded by Joseph Pilates^19^ in the 1920s.Traditional principles of Pilates exercise include centering, concentration, control, precision, flow, and breathing.^20^ The Pilates method focuses on exercises to control breathing, postural symmetry, and stabilization/flexibility of the spine, pelvis, and shoulders.^21^ It has been proved with evidence that participation in Pilates exercises can correct poor body posture, strengthen the muscles that affect poor body posture, maintain body balance, and thus alleviate spinal deformities.^22^ Lee et al,^23^ in their study of people with forward head posture, found that Pilates exercises 3 times a week for 10 weeks reduced pain and disability and improved cranial vertebral angles. Vaquero-Cristóbal et al^24^ provided evidence that Pilates was an effective way to improve hamstring extension, pelvic tilt, and maximum trunk flexion in sedentary individuals. However, some scholarly studies show that Pilates does not have a positive effect on correcting body posture.^25^

The aim of this systematic review was to explore the effects of Pilates, a low-impact form of exercise that focuses on core strength, flexibility, and body awareness,^22^ on body posture; to determine whether Pilates can be used as an effective physiotherapy exercise for the correction of postural problems; and to provide references and recommendations for subsequent research and practice by relevant physiotherapists, researchers, and health care professionals. The effects of Pilates on quality of life and pain relief are also described. A selective, purposeful, and critical review of the existing literature allows for a more comprehensive understanding of the current state of the industry. This study provided a comprehensive review of recent English language literature regarding the effect of Pilates on body posture, aiming to yield valuable insights into the effects of Pilates on body alignment and posture.

## Methods

### Search process

This systematic review was guided by the Preferred Reporting Items for Systematic Reviews and Meta-Analyses 2020,^26^ which was included in the International Prospective Registry for Systematic Reviews, CRD42023474174 (https://www.crd.york.ac.uk/prospero).

This systematic review searched 6 databases (Web of Science, PubMed, Scopus, Science Direct, Springer Link, and CNKI) for the following keywords: “Pilates” or “Pilates exercise” or “Pilates method” or “Pilates training” and “posture” or “body posture” or “poor body posture” or “postural” and “trials.” The literature search revealed a surge in research on the topic from 2018 onward, and to obtain the most recent research on the topic, we set the search years from January 1, 2019, to November 15, 2023. The literature/article search started on September 16, 2023, and ended on November 15, 2023. In addition, 2 other reviewers (F.L., R.D.O.D.) independently conducted manual searches for references to articles included in this review and references to similar reviews. This systematic review used manual checking of article titles and abstracts. Disagreements arising from the literature search were resolved by a third reviewer (K.G.S.). Table 1 shows the complete retrieval strategy.

**Table 1:** Search strategy for all databases

Databases	Search Strategy	Results
Web of Science (last 5y)	((ALL=(Pilates or Pilates exercise or Pilates method or Pilates training)) AND ALL=(posture or body posture or poor body posture or postural)) AND ALL=(trials)	41
PubMed (last 5y)	((“Pilates”[Title/Abstract] OR “Pilates exercise”[Title/Abstract] OR “Pilates method”[Title/Abstract] OR “Pilates training”[Title/Abstract]) AND (“posture”[Title/Abstract] OR “body posture”[Title/Abstract] OR “poor body posture”[Title/Abstract] OR “postural”[Title/Abstract])) AND (“trials”[Title/Abstract])	25
Scopus	TITLE-ABS-KEY: (“Pilates” OR “Pilates exercise” OR “Pilates method” OR “Pilates training” AND “posture” OR “body posture” OR “poor body posture” OR “postural” AND “trials”)	53
Science Direct (1994-2023)	Pilates or Pilates exercise or Pilates method or Pilates training and posture or body posture or poor body posture or postural and trials	236
Springer Link (2019-2023)	“Pilates” OR “Pilates exercise” OR “Pilates method” OR “Pilates training” AND “posture” OR “body posture” OR “poor body posture” OR “postural” AND “trials”	125
CNKI (last 5y)	Pilates or Pilates exercise or Pilates method or Pilates training and posture or body posture or poor body posture or postural and trials	12

### Eligibility criteria

We referred to the PICOS model in including references. PICOS is an abbreviation for the following concepts: (1) population;[(2) intervention; (3) comparison; (4) outcome; and (5) study design.^27^ Each component of PICOS was used as a criterion for inclusion in the systematic review (table 2).

**Table 2:** Inclusion and exclusion criteria were based on the PICOS model

PICOS	Inclusion Criteria	Exclusion Criteria
Population	Excluding population-specific terms, including age and sex	-
Intervention	Pilates, Mat Pilates, or a Pilates training program	Intervention did not involve Pilates
Comparison	Control group with other interventions	-
Outcome	Include posture or body posture or poor body posture	Conclusions are not related to body posture
Study design	RCT or non-RCT or quasi-experimental study	Book chapter reviews, meeting abstracts, reports, review articles

Abbreviation: RCT, randomized controlled trial.

The inclusion criteria for articles in this study were as follows: (1) ≥1 intervention was Pilates, Mat Pilates, or a Pilates training program; (2) the outcomes of the study must include the effects of Pilates on body posture or poor posture, regardless of the sex and age of the participants; (3) the study design must be a randomized controlled trial or quasi-experimental study; (4) duration of intervention was not <4 weeks; and (5) the articles were published in English.

The article exclusion criteria for this study were as follows: (1) book chapter reviews, meeting abstracts, reports, and review articles; (2) the duration of the trial intervention was <4 weeks; (3) articles did not support access to full text; and (4) non-English articles.

### Study selection

**Fig 1:**
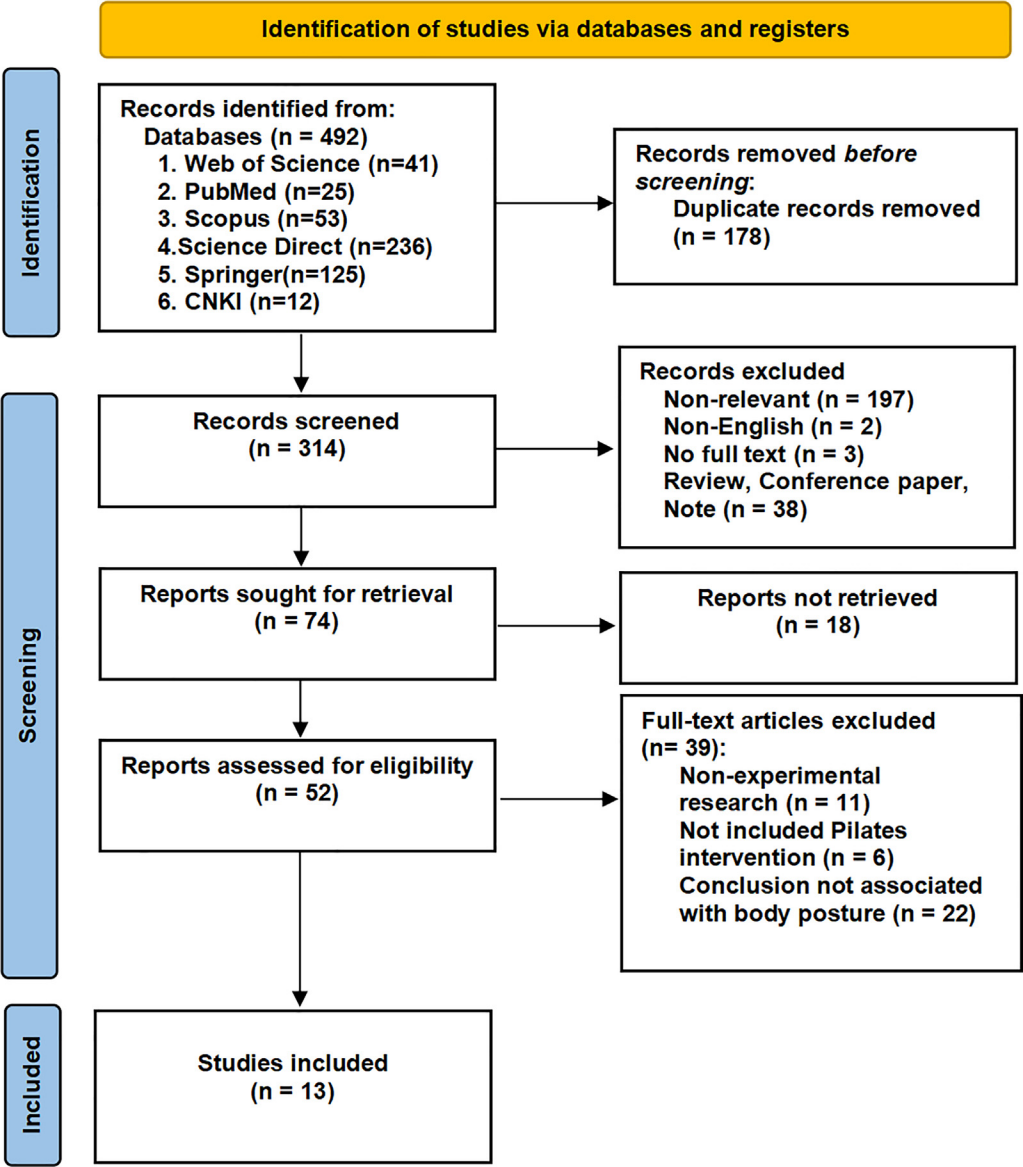
Preferred Reporting Items for Systematic Reviews and Meta-analysis 2020 flow diagram of the systematic review screening process.

The literature retrieved underwent independent assessment by 2 reviewers (F.L., C.W.) against the predefined inclusion and exclusion criteria established for this systematic review. A new database was created by the reviewers to import articles that met the inclusion criteria for this review; articles were numbered and saved after excluding duplicates. The EndNote citation management system was used to exclude duplicate articles. These steps were followed by reading the full text to screen for studies that fit into this systematic review. When disagreements were encountered during the literature assessment process, the third reviewer (K.G.S.) offered to resolve them. Figure 1 shows the review process based on the Preferred Reporting Items for Systematic Reviews and Meta-Analyses statement.

### Data extraction

Initial screening was first carried out according to the title and abstract of the literature. Next, the Discussion and Conclusion sections of the articles were read in depth. The included articles were finally analyzed and summarized in the following form: (1) author, title, year of publication, and citizenship; (2) population (sample size, control group); (3) participant characteristics (age, sex); (4) intervention characteristics (duration, frequency); and (5) study outcomes. Two reviewers (F.L., C.W.) independently included all articles in the new database using a standard coding form, and the fifth reviewer (Y.Y.) reviewed the database.

### Quality assessment and addressing the risk of bias

The Physiotherapy Evidence Database (PEDro) scale was used to determine the quality of the literature included in the review. The PEDro scale has proved to be a valuable criterion for evaluating the quality of the included literature during the construction of systematic reviews. It contains a total of 11 criteria to evaluate the internal and external validity of the article. Criteria 2-9 evaluate the internal validity of the trial, and criteria 10-11 evaluate the use of valid statistical analyses. The article quality assessment for this study was completed by a single reviewer. When a study met a criterion, that criterion was marked as YES (1 point) and conversely as NO (0 points) for statistical purposes. A study is generally scored on a scale of 0-10, with higher scores proving that the study used better research methods. Studies with a PEDro score between 8 and 10 were rated as methodologically excellent in terms of quality; those with a score between 5 and 7 were of good quality; those with a score between 3 and 4 were of moderate quality; and those with a score of <3 were of low quality.^28^

The Cochrane Risk of Bias (RoB 2.0) tool was used to assess the risk of bias for each study. Risk of bias was assessed for bias in the randomization process, bias due to deviation from the intervention, bias due to missing results, bias in the measurement of the data, and bias in the choice of reported outcomes.^29^ The internal effect risk of bias assessment for each study was completed independently by 2 reviewers (F.L., R.D.O.D.) and recorded as low risk, unclear, and high risk and marked with different color-coding systems. A third reviewer (K.G.S.) checked the results and resolved disagreements.

## Results

### Search results

Figure 1 illustrates the article search and screening process for this review. We retrieved a total of 492 relevant articles from 6 different databases. Three hundred fourteen articles remained after eliminating 178 duplicates. Again, 262 articles were excluded after reviewing the titles and abstracts of the articles. By reviewing the final 52 articles, 13 studies^8^^,^^10^^,^^30^, ^31^, ^32^, ^33^, ^34^, ^35^, ^36^, ^37^, ^38^, ^39^, ^40^ were finally included in this systematic review.

### Study quality assessment

Study quality assessment for this systematic review was conducted using the PEDro score (table 3^8^^,^^10^^,^^30^, ^31^, ^32^, ^33^, ^34^, ^35^, ^36^, ^37^, ^38^, ^39^, ^40^). Of the studies included in the review, the lowest score was 2 and the highest was 7, with most scores clustered in the range of 4-7. Of the 13 articles included in this study, 6 were of good quality, 4 were of moderate quality, and 3 were of low quality according to the criteria for evaluating article quality proposed by Kowalski et al.^3^ All articles conformed to baseline comparability. It was difficult to blind subjects, blind therapists, and blind assessors because Pilates was a specialized sport requiring specialized instruction as well as because body posture measurement was a specialized field that required a thorough knowledge base. Despite this, the researchers endeavored to make the experiment rewarding for all subjects.

**Table 3:** PEDro score

Author	Criteria											Total Score Items 2-11/10
	1	2	3	4	5	6	7	8	9	10	11	
Cibinello^30^	1	1	1	1	1	0	0	0	0	1	1	6
Pivotto^31^	1	0	0	1	0	0	0	0	0	0	1	2
Pivotto^32^	1	1	1	1	0	1	0	0	1	1	1	7
Karavelioğlu^10^	1	0	0	1	0	0	0	0	0	0	1	2
Özden^8^	1	1	1	1	0	0	0	0	0	1	1	5
Alexandru^33^	1	0	0	1	0	0	0	0	1	0	0	2
Kudchadkar^34^	1	1	0	1	0	0	0	0	0	1	1	4
de Bem Fretta^35^	1	1	0	1	0	0	0	1	1	1	1	6
Hürer^36^	1	1	0	1	0	0	0	0	1	1	0	4
González-Gálvez^37^	1	1	0	1	0	0	0	0	0	1	1	4
González-Gálvez^38^	1	1	0	1	0	0	0	1	1	1	1	6
Ozturk^39^	1	0	0	1	0	0	0	0	0	1	1	3
Niaradi^40^	1	1	1	1	0	0	0	0	0	1	1	5

NOTE. Item score: 1, meets criteria; 0, does not meet criteria; Criteria: 1, eligibility criteria; 2, random allocation; 3, concealed allocation; 4, baseline comparability; 5, blind subjects; 6, blind therapists; 7, blind assessors; 8, adequate follow-up; 9, intention to treat analysis; 10, between group comparisons; 11, point estimates and variability.

### Population characteristics

Table 4^8^^,^^10^^,^^30^, ^31^, ^32^, ^33^, ^34^, ^35^, ^36^, ^37^, ^38^, ^39^, ^40^ describes the participant characteristics of the 13 studies that met the requirements for this review; they included elementary, high school, college, adult, and elderly students, and some subjects had breast cancer, temporomandibular dysfunction (TMD), thoracic deformities, and the like.

**Table 4:** Population, intervention, and outcome

Author	Population	Intervention						Outcome
		Age, y	Sex	Type	Duration, wk	Frequency	Study Design	
Cibinello^30^	N=40, school children PG: n=20, CG: n=20	8-12	Unknown	Mat Pilates	12	2 times/wk 50 min per time	RCT	Posture→
Pivotto^31^	N=24, healthy adult women	21-35	19 FM	Pilates	15	2 times/wk 50 min per time	Quasi-experimental study	Dynamic balance↑ Static body posture↑ Posture habits↑
Pivotto^32^	N=40, women with TMD EG: n=20, CG: n=20	18-35	40 FM	Pilates	15	2 times/wk 50 min per time	RCT	Neck and back pain→ Posture→ Posture habits→
Karavelioğlu^10^	N=18, sedentary women with posture disorder	18-30	18 FM	Pilates	8	3 times/wk 90 min per time	Quasi-experimental study	Posture↑
Özden^8^	N=34, patients with adolescent idiopathic scoliosis PG: n=16, CG: n=18	15-30	5 M 29 FM	Pilates	8	2 times/wk 60 min per time	RCT(S)	Spine deformity, QoL, and perception of deformity in scoliosis→ Back and low back pain↑ Posture↑
Alexandru^33^	N=11, students with postural deviations in the sagittal plane	14-16	4M 7FM	Pilates	12	2 times/wk 50 min per time	Quasi-experimental study	Degree of curvature↑ Inversion of curvature occurs thoraco-lumbar↑
Kudchadkar^34^	N=51, asymptomatic individuals with lumbar hyperlordosis PG: n=17, EG: n=17, CG: n=17	18-40	10 M 41 FM	Mat Pilates, Egoscue exercises	4	Unknown	RCT	Hyperlordosis↑
de Bem Fretta^35^	N=34, women with breast cancer PG: n=18, CG: n=16	44-66	34 FM	Mat Pilates	16	3 times/wk 60 min per time	RCT(S)	Posture↑ Balance↑
Hürer^36^	N=46, patients with sagittal cervical disorientation PG: n=23, CG: n=23	30-60	5 M 41 FM	Clinical Pilates, home exercises	8	Unknown	RCT	Craniovertebral, head tilt, cervicothoracic angles and strength and endurance of DCF muscles↑ CROM, decreasing pain severity and functional impairment parameters→
González-Gálvez^37^	N=236, high school student EG: n=118, CG: n=118	13.15±1.24	124 M 112 FM	Pilates	36	2 times/wk 15 min per time	RCT	Averted the increase of the thoracic curvature, and decreased the curvature of the lumbar lordosis and pelvic tilt in standing position; avoided a greater increase of thoracic curvature in active alignment in standing position; and avoided the increase of thoracic curvature in trunk flexion
González-Gálvez^38^	N=103, adolescents with thoracic hyperkyphosis PG: n=52, CG: n=51	13.48±1.23	27 M 76 FM	Pilates	38	2 times/wk 15 min per time	RCT	Thoracic kyphosis in relaxed standing position↑ Hamstring extensibility↑
Ozturk^39^	N=66, preschool children PG: n=31, CG: n=35	5-6	Unknown	Pilates	10	2 times/wk	Non-RCT	Posture↑ Physical fitness parameters↑
Niaradi^40^	N=80, primary school student EG1: n=26, EG2: n=27, EG3: n=27	10-13	80 FM	Eutonia, Holistic Gymnastics, Pilates	10	1 time/wk 60 min per time	RCT(S)	Head inclination↑ Pelvic anteversion↑

Abbreviations: CG, control group; CROM, cervical range of motion; DCF, deep cervical flexors; EG, experimental group; FM, female; M, male; PG, Pilates group; QoL, quality of life; RCT(S), randomized clinical trial; RCT, randomized controlled trial.

#### Sample size

All studies included 783 subjects, the smallest of these studies had 11 subject participants, the largest had 236, and most of the studies had <50 participants, with a few in the range of 50-100.

#### Sex

The sex of the participants was indicated in 11 studies, in which 175 participants were known to be men and 502 were known to be women, and in 2 studies, the sex of the participants was mentioned. According to the results, the number of female subjects was significantly higher than that of male subjects in Pilates-related studies.

#### Age

The age distribution of the subjects ranged from 5 to 66 years, which was a very large span. Statistically, however, only 2 studies involved people >50 years of age, whereas the other subjects were generally adolescents and adults aged <30 years.

### Intervention characteristics

Table 4 describes the intervention characteristics across the studies in terms of type of intervention, duration, frequency of sessions, and study design. The primary intervention in all studies was Pilates and Mat Pilates. Three studies compared the intervention effects of Pilates with other exercises. One study compared Mat Pilates with Egoscue Exercises^34^; 1 study compared Clinical Pilates with Home Exercises^36^; and 1 study compared the workout effects of Pilates, Eutonia, and Holistic Gymnastics.^40^

In terms of Pilates intervention duration, only 1 study^34^ lasted <8 weeks, with most researchers focusing on intervention durations of 8-16 weeks, and the longest intervention durations being 36^37^ and 38^38^ weeks.

Regarding the design of the frequency of sessions, 8 studies implemented a frequency of 2 intervention sessions per week, whereas 2 other studies conducted 3 sessions per week,^10^^,^^35^ and 1 study once a week.^40^ However, 2 other studies did not mention the frequency of intervention.^34^^,^^36^

In terms of study design, 9 studies were designed as randomized controlled trials or randomized clinical trials, 3 were quasi-experimental studies,^10^^,^^31^^,^^33^ and 1 was a nonrandomized controlled trial.^39^

### Outcome

Thirteen studies on the effects of Pilates on posture were included in this review based on the inclusion criteria that we set. All the outcomes were related to body posture such as spinal deformity, spinal curvature, forward head, thoracic kyphosis, and pelvic tilt.^8^^,^^10^^,^^31^, ^32^, ^33^, ^34^, ^35^, ^36^, ^37^, ^38^, ^39^, ^40^ Additionally, some of the studies mentioned the effects of Pilates on quality of life, pain relief, and balance function in their Discussion sections.^8^^,^^10^^,^^32^

In terms of the effect of Pilates on posture, Karavelioğlu et al^10^ conducted an 8-week Pilates intervention with women who were sedentary and suffered from postural disorders, and the results showed a significant improvement in their body posture. In one of their studies, Pivotto et al^31^ illustrate that Pilates training improves static body posture during relaxation and improves postural habits in healthy adult women when picking up objects. A study on subjects with breast cancer^35^ who underwent a 16-week Mat Pilates intervention showed a significant improvement in their posture as well as in their balance function. Ozturk and Unver^39^ also demonstrated that Pilates also had a positive effect on body posture in 5- to 6-year-old preschoolers. However, Pivotto et al^32^ showed that 15 weeks of Pilates training failed to have an effective effect on body posture and postural habits in patients with TMD. In a study conducted by Cibinello et al^30^ on the effects of 3 months of Mat Pilates on posture in 8- to 12-year-olds, the results showed no significant improvement in the subjects’ posture.

In terms of the effect of Pilates on the cervical spine, in a study comparing the effects of clinical Pilates and home exercise on sagittal cervical disorders,^36^ it was shown that clinical Pilates exercises provided clinically significant improvements in craniovertebral, head tilt, cervicothoracic angles, and strength and endurance of deep cervical flexors muscles. In Niaradi et al's^40^ study of 10- to 13-year-old primary school children, Pilates exercises were found to effectively improve forward head posture, pelvic tilt, and the proper method of carrying a schoolbag.

In terms of the effect of Pilates on the thoracic spine, both studies by González-Gálvez et al^37^^,^^38^ demonstrated the significant improvement of thoracic kyphosis in adolescents in a relaxed standing position with prolonged Pilates exercise, as well as their hamstring extensibility. However, the study by Özden and Çolak^8^ showed that Pilates did not have a significant effect on spinal deformity and the perception of spinal deformity in adolescent patients with idiopathic scoliosis.

In terms of the effect of Pilates on the lumbar spine, the outcome of Kudchadkar et al's^34^ study illustrates how Pilates improved hyperlordosis in asymptomatic individuals with lumbar hyperlordosis relative to Egoscue exercises. Alexandru and George-Sebastian^33^ documented that Pilates improves both the degree of curvature and the level at which the inversion of curvature occurs in the thoracolumbar region in students with postural deviations in the sagittal plane.

Table 4 also illustrates some of the research outcomes regarding the effect of Pilates on quality of life and pain relief. The findings of a study of a Pilates intervention for adolescents with idiopathic scoliosis^8^ indicate that Pilates intervention was effective in relieving back and low back pain, but adolescents’ quality of life did not improve. However, Hürer et al's^36^ study on patients with sagittal cervical disorientation reported that Pilates did not give an advantage to patients with pain as the cervical range of motion increased. Pivotto^32^ also mentioned in the study that Pilates failed to relieve neck and back pain in patients with TMD.

We also summarized the methodology of 13 studies on the assessment of body posture (table 5^8^^,^^10^^,^^30^, ^31^, ^32^, ^33^, ^34^, ^35^, ^36^, ^37^, ^38^, ^39^, ^40^), covering mainly the measurements used, the frequency of testing, and the marking of anatomical measurement points in the subjects. By way of summary, most of the studies focused on photogrammetry, whereas the rest of the studies used computer software or related testing instruments. In terms of measurement frequency, only Pivotto^31^ took 3 measurements of subjects’ body postures, whereas the rest of the studies took 2 measurements, at baseline and posttest.

**Table 5:** Postural assessment methods

Author	Methodology	Measurement Frequency	Recording Position
Cibinello^30^	Photogrammetry	Baseline Postintervention	Glabella, TR, AC, C7, lower angle of the scapula, T3, ASIS, PSIS, major femoral trochanter, knee, medial point of the patella, tuberosity of the tibia, point on the midline of the leg, lateral malleolus, medial malleolus, point on the calcaneus tendon at the malleolus, calcaneus, and point between the head of the second and third metatarsals
Pivotto^31^	Digital Photogrammetry	30 d before the intervention started (M1) Before the intervention started (M2) Ended of intervention (M3)	CO, right TR, AC, RIAS and LIAS, RASIS and LASIS, GTRF, TLCRF, right lateral malleolus, heels, C1, C2, C4, C6, C7, T1, T2, T4, T6, T8, T10, T12, L2, L4, and S2
Pivotto^32^	Computerized Photogrammetry	Baseline Postintervention	TR, AC, SLA, CO, C1, C2 C4, C6, C7, T1, T2, T4, T6, T8, T10, T12, L2, L4, S2, PSIS, ASIS
Karavelioğlu^10^	PostureScreen Mobile (PostureCo, Inc, Trinity, FL) application	Baseline Postintervention	Bilateral pupils, sternal notch, bilateral acromioclavicular joints, bilateral T8 ribs, bilateral anterior superior iliac spines, midpoint of bilateral anterior ankles, bilateral external auditory meatus, bilateral greater trochanters of femur, bilateral inferior ear lobes, bilateral posterior superior iliac spines, bilateral posterior superior iliac spines, and bilateral Achilles’ tendons
Özden^8^	New York Posture Rating Chart test	Baseline Postintervention	T1, T2, T12, L1, S1, and S2
Alexandru^33^	Optical Capture and “Kineod” Infrared Rays	Baseline Postintervention	C7, S1, ASIS, and lower angle of the shoulder blade
Kudchadkar^34^	Observational Measurement	Baseline Postintervention	T12, S2, PSIS, and ASIS
de Bem Fretta^35^	Postural Assessment Software (SAPO) Photogrammetry	Baseline Postintervention	TR, AC, SLA, C7, T4, ASIS, PSIS, lower angle of the shoulder blade, bilateral greater trochanters of femur, and lateral malleolus
Hürer^36^	Photogrammetry	Baseline Postintervention	Craniovertebral angle is the angle between the horizontal line from C7 and the line joining C7-tragus. Head tilt angle was used to evaluate the tilt of the head in the sagittal plane. This angle is between the vertical line aligned to ear tragus and the line that joins the eye canthus and tragus. Cervicothoracic angle is the angle between tragus-C7 process line and C7-T4 spinous process line.
González-Gálvez^37^	SPINAL MOUSE SYSTEM (Idiag, Fehraltdorf, Switzerland)	Baseline Postintervention	Unknown
González-Gálvez^38^	SPINAL MOUSE SYSTEM (Idiag, Fehraltdorf, Switzerland)	Baseline Postintervention	Unknown
Ozturk^39^	sagittal plane alignment of the spine (inclinometer), posture (PostureScreen Mobile program)	Baseline Postintervention	The 13 body alignment segments include posterior views of the head, shoulders, spine, hip, feet, and arches, and lateral (left side) views of the neck, chest, shoulders, upper back, trunk, abdomen, and lower back
Niaradi^40^	Postural Assessment Software (SAPO) Photogrammetry	Baseline Postintervention	TR, AC, C7, ASIS, and PSIS

AC, acromion; ASIS, anterior superior iliac spine; C1, spinous processes of the first cervical vertebra; C2, spinous processes of the second cervical vertebra; C4, spinous processes of the fourth cervical vertebra; C6, spinous processes of the sixth cervical vertebra; C7, spinous processes of the seventh cervical vertebra; CO, occipital protuberance; GTRF, trochanter of the right femur; IAS, inferior angles of the scapulae; L1, spinous processes of the first lumbar vertebra; L2, spinous processes of the second lumbar vertebra; L4, spinous processes of the fourth lumbar vertebra; LIAS, Left inferior angles of the scapulae; LASIS, Left anterior superior iliac spine; PSIS, posterior superior iliac spine; RIAS, Right inferior angles of the scapulae; RASIS, Right anterior superior iliac spine; S2, spinous processes of the second sacral vertebra; SLA, scapula lower angle; T1, spinous processes of the first thoracic vertebra; T2, spinous processes of the second thoracic vertebra; T3, spinous processes of the third thoracic vertebra; T4, spinous processes of the fourth thoracic vertebra; T6, spinous processes of the sixth thoracic vertebra; T8, spinous processes of the eighth thoracic vertebra; T10, spinous processes of the 10th thoracic vertebra; T12, spinous processes of the 12th thoracic vertebra; TLCRF, tuberosity of the lateral condyle of the right femur; TR, tragus.

## Discussion

A total of 13 studies on the effects of Pilates on body posture were included in this systematic review, with 783 subjects participating and with no restriction on the sex of the subjects. We found that in terms of participation, Pilates is applicable to people of all ages. The findings encompassed a range of body posture issues, including but not limited to spinal deformities, forward head posture, pelvic tilt, and thoracic kyphosis. Some studies have demonstrated the positive effect of Pilates in improving posture. Some studies show no significant effect of Pilates in improving posture or spinal deformities. Owing to the differences in sample size, context, participants, and experimental design of the included studies, more research is needed to demonstrate the effect of Pilates in the area of improving body posture. In addition, some studies have mentioned that Pilates can improve the quality of life of participants and reduce pain associated with postural problems.

### Effects of Pilates on posture

Karavelioğlu^10^ demonstrated that 8 weeks of Pilates training for sedentary women was effective in improving the total posterior angle values of the participants, and they suggest that Pilates should be made more accessible to sedentary women to improve their ability to perform daily activities and improve their posture. Pivotto^31^ reported that the practice of Pilates twice a week for 30 sessions in healthy adult women improved dynamic balance, postural habits in picking up objects from the floor, and postural balance of the sagittal trunk. In addition, the body perception index of rotor spacing changed, but more research is needed to understand the effect of this change. de Bem Fretta et al^35^ reported that Mat Pilates is not a substitute for breast cancer treatment, but Pilates exercises can improve a patient's awareness of their self-posture and can improve their ability to maintain balance. Pilates can improve their center of the body and is a safe exercise practice for breast cancer survivors. Ozturk^39^ conducted a Pilates exercise program designed for 5- to 6-year-old preschoolers. Before and after the Pilates intervention, the researcher tested the participants on 13 body sizes. Measurements showed significant improvements in the participants’ body posture after the 10-week Pilates intervention, particularly in the forward rounded shoulder parameter. They also tested physical skill parameters in 5- to 6-year-old children, including Flamingo Balance, Sit and Reach, Standing Broad Jump, 30-Second Sit-Up, Bent Arm Hang, and 20-Meter Shuttle Run test scores. The results showed that Pilates also had a positive effect on physical fitness parameters in children.

However, the results of 2 other studies indicated that the Pilates intervention did not have a significant effect on the physical posture of the subject participants. Cibinello^30^ in their study centered around school students aged 8-12 years who carried out Pilates on a mat over a 12-week period, and the results showed that there was no significant improvement in the students’ posture before and after the intervention. They suggested that the reason for this phenomenon could be the enormous flexibility and mobility of the physiologically developing individual, and the postural changes in the subjects were considered to be physiological and part of growth and development. Pivotto^32^ studied the effects of Pilates on women with TMD and showed that there was no significant improvement in the participants’ static body posture and postural habits when picking up objects through the intervention, but there was a decrease in the severity of TMD in the patients in the intervention group, and they indicated that Pilates could be an effective treatment for TMD, in conjunction with muscle relaxation boards, to reduce the severity of the disease in young women.

### Effect of Pilates on the cervical spine

Hürer^36^ reported that clinical Pilates was superior to home exercise in improving postural disorders, mentioning that clinical Pilates exercises have been found to provide clinically significant improvements in craniovertebral, head tilt, cervicothoracic angles and strength, and endurance of deep cervical flexors muscles. Niaradi,^40^ in contrast, compared the effects of Eutonia, Holistic Gymnastics, and Pilates on primary school children. The study illustrated that 3 body movement practices improved the head inclination in the frontal plane and pelvic anteversion in the right and left profiles. Their study provides evidence that Pilates can be effective in improving head inclination, pelvic anteversion, and the correctness rate of carrying the schoolbag in elementary school students.

### Effect of Pilates on the thoracic spine

In a study of high school students by González-Gálvez,^37^ it was reported that after ≤36 weeks of Pilates intervention, students avoided an increase in thoracic spine curvature in the standing position and reduced lumbar lordosis and pelvic tilt curvature, avoided a further increase in thoracic spine curvature during active alignment in the standing position, and avoided an increase in thoracic spine curvature during anterior trunk tilt. In their other study^38^ of a population of adolescents with thoracic hyperkyphosis, they reported that a 38-week Pilates intervention was effective in improving thoracic kyphosis and hamstring extensibility in the relaxed state of the participants. However, Özden^8^ stated in their study that after 8 weeks of Pilates intervention training, the results showed no improvement in the spinal deformity of patients with scoliosis and their self-perception of the spine by comparing baseline and posttested angle of rotation, kyphosis and lordosis angle, and anterior shift values by the participants. Nonetheless, they suggested that although it did not improve spinal deformity in patients with scoliosis, Pilates had a positive effect on improving posture control.

### Effect of Pilates on the lumbar spine

Kudchadkar^34^ reported the effect of Pilates and Egoscue exercises on asymptomatic patients with lumbar disk herniation; the results showed that Pilates was superior to Egoscue exercises in the treatment of hyperlordosis in asymptomatic individuals with lumbar hyperlordosis. In Alexandru's^33^ study, the degree of spinal curvature and the level of change from lordosis to lordosis improved in all 11 subjects. Subjects whose values of the thoracolumbar curve differed significantly from those of the functional curve in the baseline test showed a substantial improvement in these parameters after the intervention treatment.

### The effects of Pilates on other aspects

This systematic review also provides a valuable basis in the effect of Pilates on improving quality of life and pain relief. Three of these studies^8^^,^^10^^,^^32^ illustrated that participants improved self-awareness and promoted physical health after a Pilates intervention. Subjects also gradually developed a positive attitude toward life through participation in the Pilates intervention. All of these enhanced their quality of life. Conversely, Özden^8^ showed in his study that Pilates intervention did not significantly improve the quality of life of adolescent patients with idiopathic scoliosis. This may be a result of the influence of pathological characteristics. However, the Pilates intervention was effective in improving the participants’ back and low back pain. Studies on sedentary women^10^ have mentioned that Pilates interventions can provide some relief from the fatigue and pain associated with being sedentary. In addition, Pivotto's^32^ study noted that Pilates training did not relieve neck and back pain in patients with TMD and stated that these results may be related to poor treatment adherence in the sample.

In conclusion, this systematic review provides valuable evidence in terms of the effects of Pilates on body posture. A review of the results of the 13 reviews included in this study showed that Pilates can be used by a variety of people, such as children, adolescents, middle-aged, and older adults; adolescents with idiopathic scoliosis; people with TMD; and breast cancer survivors. Pilates has a very high potential for improving spinal deformity, cervical posture, low back posture, and the pelvis. We also found that Pilates was effective in improving participants’ quality of life and relieving pain associated with poor posture. This result is consistent with the results of a recent study on the effects of Pilates on low back pain.^41^ This, to a certain extent, made their lives easier and more enjoyable and gave them a more positive attitude toward life, thus improving their quality of life and standard of living. The Pilates intervention was effective in relieving pain caused by postural distress in participants. Through the Pilates intervention, they gained core strength, spinal strength, and strength of the muscles around the neck. Postural disturbances due to muscle asymmetry were alleviated, and the stability of the cervical, spinal, lumbar, and pelvic structures was enhanced.

We also summarized how body posture has been measured in different studies, and we found that these photogrammetric methods are more commonly used in research because of the lower cost it entails. However, its disadvantage is the instability and inaccuracy of the measurement data results. Comparatively, the use of radiometric and computational measurements gives more accurate data, but the cost of the study increases.

The findings of our study are intended to provide clues for future relevant research and practice in the field of Pilates on body posture, providing evidence for a wider range of physiotherapists, health care practitioners, and researchers. We also suggest Pilates as a low-commitment, easy-to-do, high-return exercise that can be pleasurable, promote physical fitness, and enhance interpersonal interactions, and we hope that Pilates will be embraced by a wider range of people.

### Study limitations

This systematic review had some limitations. We also identified these shortcomings and tried to circumvent these limitations in future studies to provide more convincing and valuable findings.

First, there were the limitations of the retrieval strategy. There are limitations to our ability to develop a search strategy. Because of the limitation of search keywords, our search strategy may lead to the exclusion of some relevant articles. In future studies, we will develop a more comprehensive search strategy and inclusion and exclusion criteria to ensure the generalizability and comprehensiveness of the articles included in the review.

Second, the methodological quality of the studies included in the systematic review varied in terms of sample size and level of experimental design. In future research, the focus should be on selecting standardized studies with long interventions and large sample sizes to provide more favorable evidence for research in Pilates-related areas.

Third, there was the inconsistency in research outcomes. The research outcomes included in the synthesis do not wholly cover an issue, but rather scattered explanations of related issues, which resulted in a lack of in-depth research on a particular issue. In future research, the focus will be on in-depth research on a particular issue in order to gain a deeper understanding of the issue and suggest leads accordingly.

## Conclusions

The findings of this systematic review provide evidence for the role of Pilates in improving body posture problems. Pilates interventions can be applied to a wide range of people, covering children, adolescents, middle-aged, and older adults. Pilates can also be used as a complementary treatment for a number of ailments. Eight studies (61.5%) used a Pilates exercise program of 50-60 minutes twice a week, with most results showing a positive effect on posture or spinal correction. In subsequent studies, researchers can refer to this exercise frequency. However, it is still important to set the exercise frequency and duration with the actual experimental design.

In this study, Pilates was found to be advantageous in correcting spinal deformity and head forward posture relative to traditional physical therapy. Pilates has also been shown to have very high potential for improving anterior pelvic tilt and thoracic kyphosis. Not only that, Pilates has shown a positive effect in improving quality of life and relieving pain. Because Pilates is a low-cost, easy-to-do high-return exercise that can be applied to a wider range of people, it is a boon to many patients who are suffering from poor body posture problems. Pilates interventions are also recommended for wider practice by physiotherapists and health care practitioners. We will also be following up with more research and practice on Pilates in people's physical health to gain a fuller understanding of the promise and potential of Pilates in promoting people's physical health and improving their standard of living.
